# Emotion-specific vocabulary is associated with preschoolers’ emotion knowledge and behavioral emotion regulation

**DOI:** 10.1038/s41598-026-38847-3

**Published:** 2026-02-06

**Authors:** Berit Streubel, Nadia Khammous, Henrik Saalbach, Catherine Gunzenhauser

**Affiliations:** 1 Department of Educational Psychology, Leipzig University, Leipzig, Germany; 2 Leipzig University, Humboldt Science Center for Child Development (HumanKind), Leipzig, Germany; 3 Department of Psychology, Ludwigsburg University of Education, Ludwigsburg, Germany

**Keywords:** Emotion-specific vocabulary, Emotion knowledge, Emotion regulation, Preschoolers, Neuroscience, Psychology, Psychology

## Abstract

Language supports emotional development by enabling children to mentally represent conceptual emotion knowledge. Whereas previous research has linked emotion-specific vocabulary to emotion knowledge, its role in emotion regulation (ER) performance remains unclear. In this cross-sectional study we examined 197 German preschoolers aged 4–6 years (M = 5.6), all typically developing; for 10 children, parents reported exposure to an additional home language besides German. We investigated how general and emotion-specific vocabulary relate to preschoolers’ emotion knowledge (i.e., emotion recognition and knowledge of ER strategies) and their ability to regulate positive and negative emotional expressions. We further examined whether emotion knowledge mediates the link between language and ER. Results showed that, beyond general vocabulary, the size (number of words) and depth (adult-like use) of emotion-specific vocabulary interacted in predicting emotion knowledge and regulation of positive emotions. Different patterns emerged for emotion knowledge and ER. Regarding emotion knowledge, high emotion-specific vocabulary depth compensated for low size, whereas high emotion-specific vocabulary size supported regulation of positive emotions only when paired with high depth. No such effects emerged for the regulation of negative emotions. No mediating role of emotion knowledge was found. Findings suggest that a deeper understanding of emotion concepts—beyond merely knowing many emotion words—may be particularly crucial for the regulation of positive emotions.

**Supplementary Information:**

The online version contains supplementary material available at 10.1038/s41598-026-38847-3.

## Introduction

Emotion regulation (ER) is a key aspect of children’s emotional competence and a central developmental task^1^. Well-developed ER skills are linked to socio-emotional competence, prosocial behavior, good peer relationships and academic success^2–7^.

The development of ER skills is closely tied to language skills. Numerous studies report significant concurrent and longitudinal associations between children’s language competence and their ability to regulate emotions^8–16^. One proposed function of language is to enable children to adequately represent conceptual knowledge about emotions–such as related causes, consequences, and regulation strategies^17^. Recent work highlights the importance of domain-specific, i.e., emotion-specific, language for the development of conceptual emotion knowledge^18–20^. However, it remains unclear whether emotion-specific language skills contribute to children’s ER skills beyond general language skills.

Emotion knowledge refers to the conceptual understanding of emotions, including related causes, subjective feelings, physiological responses, cognitions, action tendencies and appropriate regulation strategies^20–22^. A substantial body of research links children’s general language skills (e.g., vocabulary, grammar, narrative abilities) to individual differences in emotion knowledge, such as emotion recognition and knowledge of emotion regulation strategies^20,23–28^. These associations have been observed in both preschool and grade school children, and emerge both concurrently and longitudinally^26^.

Research on cognitive development highlights the role of domain-specific language in developing domain-specific conceptual knowledge^29,30^. For instance, the acquisition of the number lexicon in early childhood supports arithmetic skills beyond small quantities^31,32^, and children’s frequent use of mental-state terms is positively related to their Theory of Mind skills, such as understanding false-beliefs^33,34^.

Similarly, in the domain of emotions it has been argued that a nuanced use of emotion words is central to the development of conceptual emotion knowledge. Emotion-specific language helps connect and categorize features such as causes, subjective experiences, and facial expressions into culturally meaningful emotion categories, thereby providing a framework for understanding, expressing, and regulating emotions^18,19,35^. Supporting this, Ogren and Sandhofer^36^ found that three-year-old children learned associations between emotional scenarios and facial expressions better when the scenarios were paired with specific emotion labels (e.g., disgusted) rather than broader labels (e.g., bad) or irrelevant information. These findings underscore the role of emotion-specific language in helping children to organize emotional information into meaningful conceptual categories. Streubel et al.^20^ demonstrated that the use of specific emotion terms may support children in organizing their knowledge about effective ER strategies. In their study with four- to nine-year-old children, they examined the relative contributions of emotion-specific and general language skills to individual differences in emotion knowledge. The authors differentiated between emotion vocabulary size (i.e., the number of words used) and emotion vocabulary depth (i.e., the degree to which children’s word usage resembles adult-like use). Because vocabulary acquisition within a specific semantic domain involves not only the number of words a child recognizes or uses, but also the precision with which word meanings are understood and differentiated from related concepts within that domain, the term vocabulary depth has been introduced to describe how closely a child’s word knowledge aligns with adult-like usage^37,38^. In this sense, adult-like usage refers to a more differentiated and context-sensitive use of emotion words. Adults typically use distinct emotion terms to describe different emotional states, even when these states are closely related (e.g., joy, pride, contentment), whereas children with less developed vocabulary depth tend to rely on a smaller set of familiar and more general emotion words (e.g., happy, good) across a range of situations, reflecting less clearly differentiated emotion concepts. Results by Streubel et al.^20^ show that children who used a broader range of emotion words–independent of their general vocabulary size–were better at recognizing facial emotion expressions and generated more adaptive strategies for regulating negative emotions. These associations were particularly evident in preschoolers (aged 4–5) but not in primary school, indicating a stronger relationship between emotion-specific language and emotion regulation knowledge in the preschool years. Interestingly, in an older age group (i.e., 8- to 9-year-olds) a larger repertoire of emotion words (size) seemed to be detrimental for one measure of emotion knowledge (i.e., knowledge of mental emotion triggers) in those children who had acquired comparatively low vocabulary depth^20^. This finding underscores the importance of considering both size and depth of emotion-specific vocabulary—and their potential interaction—to understand the relation between emotion specific language skills and conceptual development within the emotion domain. However, because the previous evidence for preschoolers was based on a relatively small sample size, replication is needed to strengthen the robustness and generalizability of these findings.

Emotion regulation refers to the process of employing specific strategies to modify the intensity, duration, or quality (e.g., valence) of emotional experiences and responses to align them with personal goals and social expectations^39–41^. Children’s general language skills are closely linked to their ER skills^8–16^. However, most studies rely on external assessments of ER, such as parents’ or teacher’s questionnaires, whereas performance-based measures remain scarce. In a longitudinal study, Vallotton and Ayoub^16^ assessed emotion regulation in 14- to 36-months-old children during home visits using standardized behavioral ratings. Expressive vocabulary, measured during a semi-structured play session, was found to predict both concurrent and later self-regulation. Similarly, Roben et al.^13^ studied children aged 18 to 48 months using a delayed waiting task to measure anger expression and regulation strategies. Vocabulary skills (indexed by mean length of utterance during naturalistic parent-child-interactions) during toddlerhood as well as their improvement over time predicted less intense and shorter anger expressions at preschool age–partly mediated by children’s use of ER strategies such as seeking support and distraction. Together, these findings highlight a close link between early language development and children’s ability to regulate emotions, especially through the use of functional strategies. Notably, these studies focus on toddlers, revealing a lack of research using performance-based ER measures when investigating the link between language and ER for this age group and beyond.

Several functions of vocabulary and language in the development of ER have been proposed^8,17^. First, language serves as a means of behavioral regulation by guiding children’s emotion-related action impulses through verbal self-instruction such as repeating rules or planning next steps. Second, it supports the use of cognitive ER strategies, for instance by enabling the verbal reappraisal of emotionally challenging situations. Third, as means of representation, language enables children to mentally organize, store, and access conceptual knowledge about emotions–such as causes, consequences, and adequate regulation strategies. Recent research highlights the crucial role of emotion-specific vocabulary in developing conceptual emotion knowledge^20,36^, raising the question of whether these skills also contribute directly to children’s ER skills. However, the unique contribution of emotion-specific vocabulary–beyond general vocabulary skills–to children’s ER skills remains unclear. From the perspective of language as a means of representation, the relationship between vocabulary and ER may be partly mediated by emotion knowledge. In other words, vocabulary may support ER indirectly by fostering a richer and more accessible conceptual understanding of emotions, which in turn facilitates the selection and retrieval of appropriate regulation strategies.

Several findings emphasize the role of emotion knowledge in children’s ER skills^42–44^. Di Maggio et al.^43^ showed that preschoolers’ emotion knowledge positively predicted ER skills, which in turn were linked to greater social competence and fewer behavioral problems. However, since ER was assessed via teacher reports, conclusions regarding children’s actual ER performance are limited. In contrast, Kromm et al.^44^ used a performance-based measure–the disappointing gift task–to assess the regulation of emotion-expressive behavior in four- to eight-year-old children. Emotion knowledge, particularly the ability to differentiate felt from expressed emotions, was positively related to children’s ability to conceal disappointment, highlighting the relevance of conceptual knowledge for effective ER. Similarly, Cole et al.^42^ found that three- to four-year-old children’s ability to generate and identify adaptive ER strategies for emotional scenarios enacted by puppets predicted their ER performance during a frustration-inducing waiting task. This suggests, that children with conceptual knowledge of ER can not only identify effective strategies but also understand when and how to apply them in emotionally challenging situations. Importantly, Cole et al.^42^ also found that children’s language skills (measured via mean length of utterance during free play) predicted the number of generated strategies. This pattern of finding suggests that language may enable children to mentally represent and articulate ER strategies which in turn may support children’s ER skills. However, to our knowledge, no study has yet directly examined whether emotion knowledge mediates the relationship between vocabulary and ER.

Previous research highlights the important role of language in children’s ability to regulate emotions^8–16^. It has been proposed that language enables the mental representation of conceptual emotion knowledge, thereby supporting the selection and implementation of appropriate ER strategies^17,35^. In particular, domain-specific language skills–namely emotion-specific vocabulary–have been shown to be positively associated with children’s emotion knowledge^20,36^. However, their role in children’s actual behavioral ER performance remains unclear. Whereas most prior research in children has focused on the regulation of negative emotions^13,15,16,42^—presumably due to their greater potential to disrupt social interactions—it is also recognized that positive emotions require regulation, for instance in situations where emotional displays may be socially inappropriate or need to be downregulated for strategic reasons^45,46^. Due to the representational function of language^17^, emotion knowledge may mediate the relationship between (emotion-specific) vocabulary and ER skills.

The present study addresses three aims: (1) to replicate findings by Streubel et al.^20^, indicating that emotion-specific vocabulary explains unique variance in preschoolers’ emotion knowledge (i.e., emotion recognition and knowledge of ER strategies) beyond general vocabulary; (2) to examine the relative contributions of general and emotion-specific vocabulary to preschoolers’ regulation of emotional expressivity (as a behavioral indicator of ER); and (3) to test whether emotion knowledge (i.e., emotion recognition and knowledge of ER strategies) mediates the relationship between children’s (general and emotion-specific) vocabulary and behavioral ER performance. To replicate and extend previous findings, we use a larger sample of preschool children with a comparable age composition, drawn from an ongoing longitudinal study. Behavioral ER performance is assessed using a computer-based paradigm designed to capture the downregulation of both negative and positive emotion-expressive behavior. We hypothesize that (1) emotion-specific vocabulary predicts emotion knowledge (i.e., emotion recognition and knowledge of ER strategies) above and beyond general vocabulary, (2) emotion-specific vocabulary also predicts regulation of emotional expressivity beyond general vocabulary and (3) the relation between vocabulary and regulation of emotional expressivity is mediated by emotion knowledge. Additionally, we explore whether emotion-specific vocabulary size and depth each account for unique variance in emotion knowledge (i.e., emotion recognition and knowledge of ER strategies) and emotion regulation, and whether their interaction further explains individual differences in these competencies.

## Results

### Descriptive data

Descriptive statistics for all main variables are shown in Table 1. Table 2 shows bivariate associations between main variables and potential covariates. Pearson coefficients were used for all correlations, except for those involving both ER indices, for which Spearman coefficients were used due to violation of the normal distribution. Bivariate correlations revealed positive associations between all measures of general and emotion-specific vocabulary and emotion knowledge (i.e., emotion recognition and knowledge of ER strategies). Age was positively related to general vocabulary and both emotion knowledge subscales. A significant negative relation emerged between gender and emotion recognition, with boys scoring lower on this measure. Parental education was positively related to children’s general vocabulary.

**Table 1:** Descriptive data (means, standard deviations, minimums, and maximums) for all main variables.

	Scale	M (SD)	Min.	Max.
General vocabulary	0–40	16.0 (8.3)	0	35
Emotion-specific vocabulary				
Size	0–20	5.35 (1.6)	1	10
Depth	0–5.00^a^	1.25 (0.5)	0.13	2.46
Emotion knowledge				
Emotion recognition	0–10	7.95 (1.9)	0	10
Knowledge of ER strategies	0–12	7.75 (2.8)	0	12
Regulation of emotional expressivity^b^				
Negative	–	− 1.29 (0.99)	− 9.57	− 0.29
Positive	–	− 1.92 (4.17)	− 32.5	0

^a^A participant reaches the scale maximum when s/he uses the word for each vignette that has been produced most frequently by adults.

^b^Regulation of emotional expressivity was calculated as the ratio of negative or positive expressivity in the regulation condition over the non-regulation condition and multiplied by − 1. Values greater than − 1 indicate a reduction in expressivity from the non-regulation to the regulation condition, whereas values smaller than − 1 indicate an increase in expressivity.

**Table 2:** Bivariate correlations between main variables, age group, gender, and parents’ education.

	1	2	3	4	5	6	7	8	9	
1. General vocabulary	–									
Emotion-specific vocabulary										
2. Size	0.284^***^	–								
3. Depth	0.275^***^	0.440^***^	–							
Emotion knowledge										
4. Emotion recognition	0.299^***^	0.321^***^	0.455^***^	–						
5. Knowledge of ER strategies	0.168^*^	0.214^***^	0.218^***^	0.264^***^	–					
Regulation of emotional expressivity										
6. Negative	− 0.037	0.002	0.013	0.021	0.003					
7. Positive	0.047	− 0.044	0.073	0.039	0.088	− 0.057				
8. Age	0.149^*^	0.122	0.107	0.245^***^	0.203^**^	− 0.094	− 0.097	--		
9. Gender	− 0.033	− 0.116	− 0.031	− 0.188^**^	− 0.021	0.036	− 0.135	0.082		
10. Parents’ education	0.276^***^	0.104	0.131	0.088	0.006	0.142	− 0.005	0.008	− 0.021	

Coding of Gender: 1 = female, 2 = male. Coding of parents’ education: 0 = no parent has a college degree, 1 = at least one parent has a college degree. Two-tailed Pearson correlation coefficients were used for all correlations, except for those involving both regulation of emotional expressivity indices, for which two-tailed Spearman coefficients were used.

*p ≤ .05.

**p ≤ .01.

***p ≤ .001

### Preliminary analyses

To test children’s responsiveness to the regulatory instruction in the regulation condition of the Balloons Game, a repeated measures ANOVA was conducted on the raw expressivity scores with condition (non-regulation vs. regulation) and valence of expressivity (positive vs. negative) as within-subject factors, and gender as between-subject factor. This analysis examined whether children’s emotional expressivity differed between the regulation and non-regulation condition for both positive and negative emotions. Results revealed significant main effects of condition, *F*(1,195) = 11.26, *p* < .001, *η*^2^ = 0.06, and valence *F*(1,195) = 8.72, *p* = .004, *η*^2^ = 0.01, qualified by a significant condition-by-valence interaction, *F*(1,195) = 14.79, *p* < .001, *η*^2^ = 0.07. Post-hoc paired two-tailed *t*-tests showed a significant reduction in positive expressivity from the non-regulation (*M* = 0.06, *SD* = 0.06) to the regulation condition (*M* = 0.044, *SD* = 0.053), *t*(196) = 3.77, *p* < .001. In contrast, negative expressivity did not significantly differ between the non-regulation (*M* = 0.039, SD = 0.028) and regulation condition (*M* = 0.041, SD = 0.028). Additionally, a significant valence-by-gender interaction, *F*(1,195) = 5.52, *p* = .020, *η*^2^ = 0.03, indicated that girls exhibited greater positive expressivity than boys across both conditions (*M*_*girls*_ = 0.061, *M*_*boys*_ = 0.044), *F*(1,195) = 5.87, *p* = .016, *η*^2^ = 0.03. No significant gender differences were observed for negative expressivity (*M*_*girls*_ = 0.039, *M*_*boys*_ = 0.041). Inspection of individual data revealed that 44% of children showed reduced negative expressivity in the regulation condition, whereas 56% did not. For positive expressivity, 61% of children showed reduced expression in the regulation condition, whereas 39% did not. These descriptive data indicate that the Balloon Game captures inter-individual variability in emotion regulation, even when group-level changes in negative expressivity are small.

### Contributions of emotion-specific and general vocabulary to emotion knowledge

To test Hypothesis 1—that children’s emotion-specific vocabulary explains unique variance in emotion knowledge (i.e., emotion recognition and knowledge of ER strategies) beyond general vocabulary—we conducted stepwise multiple regression analyses. Given the observed associations of age and, in the case of emotion recognition, gender with the dependent measures, these variables were included as covariates in the respective models. In the first step (Model 1 in Table 3), we examined contributions of general vocabulary to children’s emotion knowledge. Findings revealed significant positive associations with both emotion knowledge subscales. To assess the incremental value of emotion-specific vocabulary, we added either emotion-specific vocabulary size (Model 2a), emotion-specific vocabulary depth (Model 2b), or both (Model 3) as predictors. Finally, an interaction term between emotion-specific vocabulary size and depth was included (Model 4) to explore whether the effect of one measure depends on the level of the other.

**Table 3:** Stepwise multiple regression analyses predicting emotion recognition and knowledge of emotion regulation strategies from general and emotion-specific vocabulary.

Predictors (z-standardized)	Emotion recognition	Knowledge of ER strategies
Model 1		
Age	0.421***	0.518**
Gender	− 0.373**	–
General vocabulary	0.490***	0.400*
*R* ^2^	0.169	0.061
*F*	13.1***	6.27**
Model 2a		
Age	0.383**	0.480*
Gender	− 0.325**	–
General vocabulary	0.380**	0.272
Emotion-specific vocabulary size	0.415***	0.471*
*R* ^2^	0.213	0.086
*F*	10.6***	6.04***
Model 2b		
Age	0.370**	0.485*
Gender	− 0.352**	–
General vocabulary	0.299*	0.270
Emotion-specific vocabulary depth	0.728***	0.492*
*R* ^*2*^	0.306	0.088
*F*	21.1***	6.23***
Model 3		
Age	0.360**	0.466*
Gender	− 0.337***	–
General vocabulary	0.274*	0.215
Emotion-specific vocabulary		
Size	0.152	0.329
Depth	0.669***	0.364
*R* ^2^	0.311	0.099
*F*	17.2***	5.26***
Model 4		
Age	0.348**	0.453*
Gender	− 0.362***	–
General vocabulary	0.285*	0.225
Emotion-specific vocabulary		
Size	0.128	0.310
Depth	0.560***	0.266
Size × depth	− 0.400***	− 0.361^+^
*R* ^2^	0.358	0.116
*F*	17.6***	5.0***

Coding of Gender: 1 = female, 2 = male. Standardized regression coefficients (*β*), *R*^2^, F-statistics, and significance levels are reported. ^+^*p* ≤ .055. *p ≤ .05. **p ≤ .01. ***p ≤ .001.

**Fig. 1:**
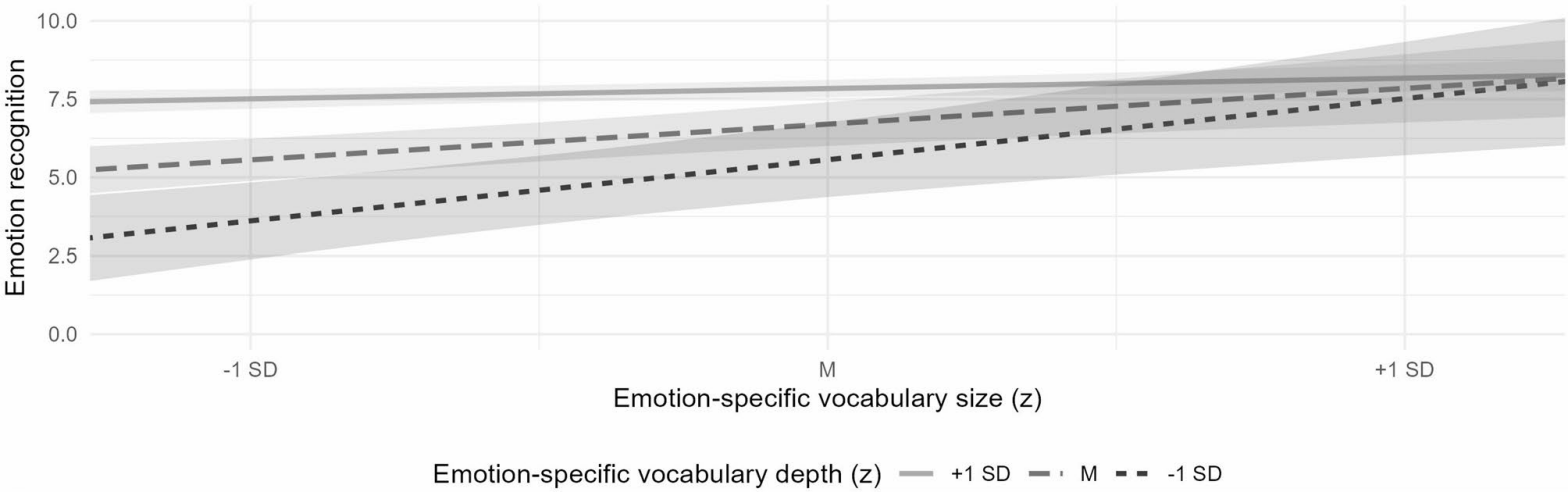
Simple slopes of emotion-specific vocabulary size on emotion recognition as a function of emotion-specific vocabulary depth. The effects are demonstrated at one standard deviation below the mean (− 1 SD), at the mean (M) and one standard deviation above the mean (+ 1 SD) of vocabulary depth. The 95% confidence intervals are represented by bands demarcating the lines.

Findings revealed that both the size and the depth of children’s emotion-specific vocabulary significantly and positively predicted emotion recognition and knowledge of emotion regulation strategies, over and above general vocabulary (Models 2a and 2b). When both predictors were entered simultaneously (Model 3), only depth remained a significant predictor of emotion recognition, whereas neither significantly predicted knowledge of emotion regulation strategies. This pattern held when the interaction term was added (Model 4). Importantly, a significant interaction emerged between emotion-specific vocabulary size and depth in predicting emotion recognition. Building on previous research indicating that an imbalance in the development of emotion-specific vocabulary size and depth may affect other aspects of emotional development^20^, we further examined this interaction effect by testing whether the relation between emotion-specific vocabulary size and emotion recognition depends on emotion-specific vocabulary depth. To this end, we conducted simple slopes analyses based on the linear regression model including the interaction term. Using the model coefficients and variance–covariance matrix, we calculated the conditional effects of vocabulary size at three levels of vocabulary depth (one standard deviation below the mean, at the mean, and one standard deviation above the mean), and tested their statistical significance using t-tests. Findings revealed that emotion-specific vocabulary size positively predicted emotion recognition only at low levels of vocabulary depth (*B* = 0.528, *SE* = 0.161, *t* = 3.28, *p* = .001), but not at average (*B* = 0.128, *SE* = 0.126, *t* = 1.02, *p* = .310) or high levels (*B* = − 0.272, *SE* = 0.169, *t* = − 1.61, *p* = .110). Visual inspection of the simple slopes (Fig. 1) indicates that the positive association between emotion-specific vocabulary size and emotion recognition decreases as emotion-specific vocabulary depth increases, suggesting that a larger emotion-specific vocabulary supports recognition particularly when children’s understanding of the underlying concepts is still limited.

**Fig. 2:**
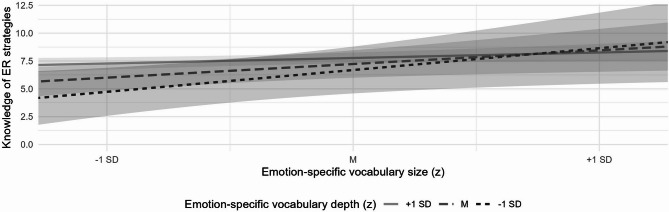
Simple slopes of emotion-specific vocabulary size on knowledge of emotion regulation strategies as a function of emotion-specific vocabulary depth. The effects are demonstrated at one standard deviation below the mean (− 1 SD), at the mean (M) and one standard deviation above the mean (+ 1 SD) of vocabulary depth. The 95% confidence intervals are represented by bands demarcating the lines.

The interaction effect of emotion-specific vocabulary size and depth on children’s knowledge of emotion regulation strategies was marginally significant (*p* = .055). Nevertheless, we explored simple slopes based on the linear regression model including the interaction term to examine whether the pattern resembles the interaction found for emotion recognition. Using the model coefficients and variance–covariance matrix, we calculated the conditional effects of vocabulary size at three levels of vocabulary depth (1 SD, M, + 1 SD) and tested their statistical significance using t-tests. This analysis revealed a very similar pattern: emotion-specific vocabulary size positively predicted knowledge of emotion regulation strategies only in children with vocabulary depth at least 1 standard deviation below the mean (*B* = 0.667, *SE* = 0.283, *t* = 2.37, *p* = .019; Fig. 2).

### Contributions of emotion-specific and general vocabulary to regulation of emotional expressivity

**Fig. 3:**
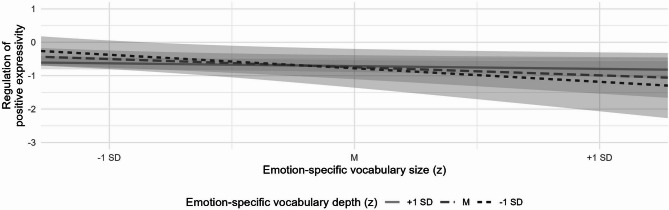
Simple slopes of emotion-specific vocabulary size on the regulation of positive expressivity as a function of emotion-specific vocabulary depth. The effects are demonstrated at one standard deviation below the mean (− 1 SD), at the mean (M) and one standard deviation above the mean (+ 1 SD) of vocabulary depth. The 95% confidence intervals are represented by bands demarcating the lines.

To test Hypothesis 2–that children’s emotion-specific vocabulary explains variance in the regulation of positive and negative expressivity beyond general vocabulary–we conducted stepwise multiple regression analyses. Due to the lack of normal distribution in both ER indices, robust regression analyses were employed to reduce outlier influence. For the regulation of positive expressivity, general vocabulary did not significantly predict outcomes (Model 1, Table 4). Similarly, neither size nor depth of emotion-specific vocabulary were significant predictors when added separately (Model 2a and 2b) or together (Model 3). However, after including the interaction term, size and depth of emotion-specific vocabulary interacted significantly in predicting regulation of positive expressivity (Model 4). To further investigate this interaction, we calculated simple slopes of emotion-specific vocabulary size at three levels of vocabulary depth (− 1 SD, mean, + 1 SD) based on the robust regression model. Standard errors, *t*-values, and *p*-values were obtained from the model’s robust covariance matrix. Emotion-specific vocabulary size showed a marginally significant negative effect on regulation of positive expressivity at low levels of vocabulary depth (*B* = − 0.122, *SE* = 0.070, *t* = − 1.75, *p* = .082) but not at average (*B* = − 0.043, *SE* = 0.055, *t* = − 0.780, *p* = .436) or high levels (*B* = 0.009, SE = 0.066, t = 0.543, *p* = .588). A visual inspection of the simple slopes suggests that a large emotion-specific vocabulary size may impede children’s ability to regulate positive expressivity, whereas a pronounced depth of vocabulary seems to compensate for this effect (Fig. 3). Regarding the regulation of negative expressivity, none of the predictors—general vocabulary, emotion-specific vocabulary size, depth, or the interaction term between size and depth—showed significant effects in any of the tested models (Table 4).

**Table 4:** Stepwise multiple regression analyses predicting children’s regulation of emotional expressivity from general and emotion-specific vocabulary.

Predictors (z-standardized)		Regulation of positive expressivity	Regulation of negative expressivity
Model 1			
General vocabulary		− 0.006	− 0.021
*R* ^2^		0.000	0.002
Model 2a			
General vocabulary		0.007	− 0.022
Emotion-specific vocabulary size		− 0.043	0.006
*R* ^2^		0.004	0.002
Model 2b			
General vocabulary		0.002	− 0.021
Emotion-specific vocabulary depth		− 0.028	0.002
*R* ^2^		0.002	0.002
Model 3			
General vocabulary		0.009	− 0.022
Emotion-specific vocabulary			
Size		− 0.038	0.006
Depth		− 0.013	0.000
*R* ^2^		0.004	0.002
Model 4			
General vocabulary		0.002	− 0.021
Emotion-specific vocabulary			
Size		− 0.043	0.001
Depth		0.016	− 0.012
Size × depth		0.079*	− 0.036
*R* ^2^		0.019	0.008

Standardized regression coefficients (*β*), *R*^2^ values, and significance levels from robust M-estimator regressions (Huber weighting, robust standard errors) are reported. *p ≤ .05.

### Emotion knowledge as a mediator of the relationship between language and regulation of emotional expressivity

The findings obtained from the testing of Hypothesis 1 and 2 confirmed one requirement for a potential mediation of the relationship between children’s language skills and their ability to regulate emotion- expressive behavior through emotion knowledge (i.e., emotion recognition and knowledge of ER strategies): significant associations between children’s (general and emotion-specific) vocabulary (independent measures) and their emotion recognition (proposed mediator). In line with current perspectives on mediation analysis^47–49^, we did not consider a significant total effect (i.e., a direct association between vocabulary and ER indices) as a necessary requirement for testing mediation.

To examine whether emotion recognition qualifies as mediator, we conducted regression analyses with children’s regulation of positive and negative expressivity as dependent measures and emotion recognition as predictor. Given the non-normal distribution of the ER indices, robust estimation was used to reduce the influence of outliers. Results revealed no significant association between emotion recognition and ER positive (*β* = − 0.011; *p* = .684) or ER negative (*β* = − 0.010.; *p* = .587). Because this essential condition for mediation was not met, no further mediation analyses were conducted to test Hypothesis 3.

## Discussion

Previous research has revealed that children with well-developed language skills tend to outperform their peers with regard to emotion knowledge^20,23–28^, and that they might also be better able to regulate emotions^8–16^. There is also some evidence that emotion-specific vocabulary contributes to emotion knowledge beyond general vocabulary, particularly in preschoolers^20^.

In a first step, we aimed to replicate the findings reported by Streubel et al.^20^, investigating the contributions of general and emotion-specific vocabulary to children’s emotion knowledge. We assessed two indicators of emotion knowledge, emotion recognition and knowledge of emotion regulation strategies. In line with Streubel et al.^20^, general vocabulary significantly predicted emotion knowledge in the models that did not control for children’s emotion-specific vocabulary. Moreover, our findings revealed that both size and depth of children’s emotion-specific vocabulary significantly and positively predicted their emotion knowledge beyond general vocabulary. In contrast to Streubel et al.^20^, our results show that general vocabulary remained a significant predictor of emotion recognition (but not knowledge of emotion regulation strategies) even after taking emotion-specific vocabulary into account.

In addition to the analyses reported in previous research^20^, we conducted an analysis of the interaction between children’s emotion-specific vocabulary size and depth in predicting emotion knowledge (i.e., emotion recognition and knowledge of ER strategies). The findings revealed a significant interaction effect with regard to emotion recognition. Whereas the interaction effect for knowledge of emotion regulation strategies was only marginally significant, the directional pattern of the coefficients was similar to that observed for emotion recognition. A closer examination of interaction effects revealed that for children with a small emotion-specific vocabulary size, depth made a difference to their emotion knowledge. Children with a small but deep emotion-specific vocabulary had emotion knowledge (i.e., emotion recognition and knowledge of ER strategies) similar to peers with a large vocabulary. However, those with a small emotion-specific vocabulary and a low vocabulary depth showed less emotion knowledge. Thus, emotion-specific vocabulary depth seems to compensate for poor emotion-specific vocabulary size with regard to children’s emotion knowledge, reflecting children’s ability to differentiate related emotion concepts and apply them appropriately across contexts: Children who have relatively few emotion words in their active vocabulary, but use those words in a more adult-like and precise manner, may be better able to identify the expressive cues associated with those emotions (and potentially to generate adaptive emotion-regulation strategies) than peers who use the same words in a more vague or unspecific way. Notably, for children with a large emotion-specific vocabulary size, a low emotion-specific vocabulary depth did not have a detrimental effect on their emotion knowledge. This seems surprising, since previous research indicates that knowing many emotion words without understanding the semantic boundaries among them might rather hinder a precise conceptual representation of emotions^20^. Further research is needed in order to investigate replicability of this finding. Taken together, findings of the study by Streubel et al.^20^ were overall replicated. Children’s emotion-specific vocabulary contributed to emotion knowledge beyond general vocabulary. Moreover, the present study indicated that children’s emotion-specific vocabulary size and depth interact in their influence on emotion knowledge (particularly emotion recognition), with emotion-specific vocabulary depth compensating for poor emotion-specific vocabulary size.

Language skills may support not only emotion knowledge but also emotion regulation performance in emotion-eliciting situations^8–17^. Specifically, it has been suggested that emotion-specific vocabulary supports the representation of emotional concepts^18,19,35^, including knowledge about adaptive strategies to regulate these emotions^20^. However, previous studies primarily focused on general language skills^8–16^. In a second step, we therefore examined the relative contributions of general and emotion-specific vocabulary to preschoolers’ ER performance. In order to investigate ER performance, we adopted an experimental design in which children played a computer game that involved trying to pop as many floating balloons as possible by clicking on them^50,51^. The game was designed to include prototypical negative situations – being prevented from popping the balloons by a dysfunctional computer mouse, and getting the feedback of having failed – and prototypical positive situations, namely being celebrated for having won the game^50,51^. Children were told to make sure that an observer could not tell whether they were winning or losing, thus targeting the regulation of positive and negative emotional expressivity within the same paradigm. Expressivity was videotaped and analysed using automated coding (FaceReader)^52^. ER performance was calculated as the ratio of children’s emotional expressivity between a non-regulation and a regulation condition. There were no significant effects of general or emotion-specific vocabulary on regulation of negative expressivity, whereas there were some effects for the regulation of positive expressivity.

For the regulation of positive expressivity, neither general vocabulary nor emotion-specific vocabulary size or depth showed direct effects on individual differences in ER performance. However, emotion-specific vocabulary size and depth significantly interacted. An investigation of the simple slopes indicated that this interaction effect might primarily be driven by a negative association between children’s emotion-specific vocabulary size and their ability to regulate positive expressivity for children with low emotion-specific vocabulary depth, although this negative association was marginally significant only. However, higher emotion-specific vocabulary depth seemed to compensate for this disadvantage. Thus, knowing many emotion words might be even detrimental to effective ER unless combined with a more adult-like understanding of the semantic boundaries of those emotion words. Emotion regulation requires understanding of social and situational demands as well as selection of adaptive and effective strategies. Although children in the present study were given a clear regulatory aim–concealing from an onlooker whether they were winning and losing–they still had to identify what this would mean for their intended emotional expressions (e.g., trying to maintain a “poker face” or trying to look consistently happy throughout the game). In a next step, they were required to select and implement one of several possible emotion regulation strategies, e.g., response-focused suppression, masking, or cognitive strategies such as mental distancing or reappraisal, which might also be a way to influence emotional expression. A large, but unspecific and vague emotion-specific vocabulary might not be helpful, but rather overwhelming when forced to select an emotion-regulation strategy. In contrast, higher emotion-specific vocabulary depth–that is, a more adult-like understanding and usage of emotion words–seems to help children with large vocabulary size to correctly perceive the situational emotion regulation demands and select appropriate strategies^20^.

Since our theoretical framework does not suggest that the associations between emotion-specific vocabulary and emotion regulation should differ by emotional valence, factors related to the measurement of emotional expressivity may offer a more plausible explanation for the absence of effects for negative expressivity regulation. Although the non-regulation condition did not involve an instruction to regulate emotions, children’s emotional expressions in this condition might have been influenced by culturally shaped display rules. Children might have felt comfortable expressing joy quite freely when winning, but emotion socialization experiences might have prevented them from displaying unregulated negative emotion when failing^53^. This is in line with our finding that, on the group level, there was no significant difference in negative (as opposed to positive) expressivity between the non-regulation and regulation condition. Relatedly, variance in the down-regulation of negative expressivity was descriptively smaller than the variance in the down-regulation of positive expressivity. Moreover, recent research on temporal dynamics of emotion regulation in children indicate that children’s reactions to negative stimuli—and their corresponding regulatory efforts— may be measured more effectively on a finer time scale^54^. To sum up, the present study did not find support for direct associations between either general or emotion-specific vocabulary and children’s ER performance, but indicated an interactive dynamic between emotion-specific vocabulary size and depth. Specifically, the association between children’s emotion-specific vocabulary size and the ability to regulate positive emotions seemed to depend on emotion-specific vocabulary depth, the latter showing a protective effect.

Finally, we aimed at investigating whether children’s emotion knowledge (i.e., emotion recognition and knowledge of ER strategies) might mediate the association between their general and emotion-specific vocabulary and their ER performance. Although previous research indicates links between children’s vocabulary skills and their ER performance^13,16^, as well as between emotion knowledge and ER performance^42,44^, the latter associations were not found in the present study. Consequently, mediation analyses were not conducted. One possible explanation for this finding is that the IDS emotion regulation subscale only assesses strategy knowledge for regulating negative emotions (anger, fear, sadness), whereas our experimental task captured regulation of both negative and positive emotions. Moreover, our emotion knowledge measure focused on emotion recognition and emotion regulation strategy knowledge. Future studies should include further aspects of emotion knowledge, such as children’s knowledge about the difference between emotional experience and expression^44^. Another explanation relates to the nature of our ER assessment. It should be noted that emotion regulation may differ depending on the specific emotion involved and the perceived need to regulate it. However, the present paradigm focused on children’s ability to behaviorally modulate emotional expressivity under standardized regulatory demands, rather than on emotion-specific regulatory goals or strategies. Thus, our measure included prototypical situations to elicit positive and negative emotions, allowing for an objective, performance-based assessment of children’s emotion-expressive behavior, but it did not provide insights into their emotional experiences and regulatory intentions. Thus, children might have differed in their actual emotional experiences and in their perceived need to regulate. Future studies should differentiate between specific emotional experiences on the individual level and address the perceived need to regulate emotions in order to more fully capture the role of emotion knowledge as a potential mediator.

Like any empirical study, the present research has several limitations. Firstly, the cross-sectional design precludes any conclusion about the directions of effects. Although theoretical considerations suggest causal associations between children’s vocabulary, emotion knowledge, and ER performance, future studies should use longitudinal designs to better clarify the plausible direction of effects. Secondly, participants in our study were not representative for children in Germany, with an overrepresentation of highly educated parents and an underrepresentation of dual language learners. Although theoretical considerations do not suggest that associations between children’s emotion-specific vocabulary, emotion knowledge and ER performance should differ based on these background variables, the relatively homogeneous nature of our sample might have resulted in limited variability in the constructs assessed. Thus, caution is warranted regarding the generalization of findings. Thirdly, there were limitations regarding the measurement of emotion-specific vocabulary and emotion knowledge. On the one hand, the complexity and valence of the emotions assessed differed between our measures of emotion-specific vocabulary and emotion knowledge. This mismatch may have contributed to some of the findings observed and should be addressed in future research by using instruments that are more aligned. On the other hand, both the emotion-specific vocabulary measure and the emotion recognition measure required children to name emotions based on facial expressions of emotions. Although the emotion-specific vocabulary task used facial expression only as a part of a rich description of an emotional episode, this might have resulted in some overlap between the measures. Fourthly, the ER task did not reveal significant changes in children’s negative expressivity from the non-regulation to the regulation condition. Thus, our measure may not have captured successful downregulation of negative expressivity at the group level. Although our analyses focused on individual differences rather than overall success in ER, this limitation may partly explain the absence of effects for children’s regulation of negative expressivity. Finally, it should be noted that children’s emotion knowledge and emotion regulation are likely influenced by a multitude of factors beyond the ones focused and controlled for in the present study, for instance, by parental socialization and executive functions^8^. Future studies should aim at a more comprehensive statistical model integrating these factors.

The present study adds to previous findings showing that emotion-specific vocabulary contributes to children’s emotion knowledge above and beyond general vocabulary. Importantly, findings suggest that emotion-specific vocabulary size and depth do not influence children’s emotional competence merely in an additive way, but interact in explaining individual differences in emotion knowledge and in the regulation of positive emotions. Specifically, emotion-specific vocabulary depth might buffer against the detrimental effect of poor emotion-specific vocabulary size with regard to emotion knowledge. Moreover, the regulation of positive emotional expressions in children with large emotion-specific vocabulary might be impaired when combined with low emotion-specific vocabulary depth. From a practical point of view, the present study adds to the evidence that emotion-specific vocabulary might be a starting point when aiming at fostering children’s emotional competence^55^. In particular, our findings suggest that parents and professionals should carefully consider not only how many emotion words children use, but whether they grasp the semantic boundaries of the associated emotion concept. Future studies should continue to investigate indirect pathways linking language skills, emotion knowledge, and emotion regulation performance.

## Methods

### Participants

The data for the present study were collected as part of an ongoing longitudinal study on the development of language and emotional competencies from preschool to elementary school age. At the first measurement point, 301 preschoolers aged 4 to 6 years (*M* = 5.6, SD = 0.4; 48.5% girls) participated. The current subsample (*N* = 197; *M* = 5.6, SD = 0.4; 47.7% girls) includes those children of the initial sample who completed the emotion regulation task. Participants were recruited from a mid-sized German city via a database of families who had provided written consent to participate in developmental psychology studies. For 10 children, parents reported that an additional language besides German was regularly spoken at home; however, given the small number, this variable was not included in the analyses. No children with diagnosed language disorders were reported. Parental education data were available for 135 children; 64.4% of these had at least one parent with a university degree. Nationally representative data from Germany indicate substantially lower rates of parental academic education: according to the Social Report 2024^56^, only 32% of students had at least one parent with a university degree. This indicates that the present sample is characterized by a disproportionately high parental education level. Due to sensitivities in demographic data collection in Germany, no further data on ethnicity, race, or socioeconomic status were collected. According to the official statistics, the population from which participants were drawn was 87.1% native Germans and predominantly middle class^57^. The study was approved by the Ethics Committee of the Medical Faculty of the University of Leipzig (Ethik-Kommission an der Medizinischen Fakultät der Universität Leipzig) and conducted in accordance with the Declaration of Helsinki and relevant German psychological research regulations.

### Procedure

Children were individually tested by trained investigators in a quiet room at their preschool across two sessions of up to 45 min. The first test session included measures of general and emotion-specific vocabulary and emotion knowledge (i.e., emotion recognition and knowledge of ER strategies). The second session, conducted 1 to 14 days later, included the ER task, a grammar test, and a measure of executive self-regulation (the latter two not reported here). Test order within each session was fixed to minimize potential interference or facilitation effects.

### Measures

#### General expressive vocabulary

 General vocabulary was assessed using the short version of the WWT 6–10^58^, a standardized picture naming test normed for children age 5;6 to 6;11. The test included 40 colour-photo items targeting nouns, verbs, categorical nouns, and adjectives. Because some participants were younger than the norming age, a discontinuation rule was applied for categorical nouns and adjectives–item types with lower success rates in 5- to 6-year-olds^58^, in order to avoid overtaxing the children. The discontinuation rule was enacted when three consecutive items were unanswered. This rule was applied in 83 cases. Each correct response yielded one point (max. 40). Raw scores were used in all analyses.

#### Emotion-specific vocabulary

 Emotion-specific vocabulary was assessed using the Children’s Emotion-specific Vocabulary Vignettes Test^20,59^, comprising 20 short vignettes with drawings and audio-recorded narrations. Each vignette depicts a child protagonist experiencing a specific emotional state, showing facial expressions, physiological reactions, and appraisals. The 20 vignettes feature 6 basic emotions (joy, fear, sadness, anger, disgust, and surprise) as well as 14 complex emotions that are semantically related but subordinated to the basic emotion categories (e.g., pride and contentment as subordinated emotion concepts of joy; frustration and envy as subordinated emotion concepts of anger; disappointment, loneliness, and yearning as subordinated emotion concepts of sadness)^60^. Presented on a tablet, children were asked to name the emotions of the protagonist. Following a standardized procedure, children were encouraged to name the emotion that best described the protagonist’s emotional state. If they responded with an unspecific emotion label (e.g., ‘well’ or ‘bad’), they were asked to clarify (‘Can you describe it more precisely?’). If they described looks or actions (e.g., laughing or crying), they were prompted to name the accompanying feeling (‘How do you feel when you …?’). If children provided several emotion labels at the same level of specificity (e.g., multiple basic emotions or multiple complex emotions), they were asked to indicate which emotion best described the protagonist’s feeling. Conversely, if children provided emotion labels at different levels of specificity the more specific term was retained without further prompting (e.g., ‘happy’ over ‘good’, or ‘proud’ over ‘happy’). This procedure ensured that one semantically most specific and contextually appropriate emotion label per vignette was retained for scoring purposes. This approach was chosen to obtain a standardized, performance-based measure of emotion-specific vocabulary under comparable elicitation conditions across children. Children’s responses were spell-checked and automatically coded using EmotionTool 1.0 (ET1.0)^61^, a Python-based program that identifies emotion words from a pre-defined list. Emotion-specific vocabulary *size* was measured as the number of different emotion words used across all vignettes (max. 20). *Depth* was determined as the degree to which each child’s emotion words use corresponded to the naming pattern produced by the parent sample of the present study across the 20 vignettes. The parent sample (*N* = 238) produced a larger and more semantically differentiated set of emotion labels, including more specific and subordinate terms, resulting in higher mean size (*M* = 14.63, *SD* = 2.64) and depth scores (*M* = 3.18, *SD* = 0.66) than the child sample (descriptive statistics for the child sample are reported in the Results section). To quantify emotion-specific vocabulary depth, for each vignette a similarity score was calculated that integrated three components: (a) a frequency score (range 0–1) reflecting how often the child’s chosen label was used by adults for the same vignette; (b) a small base score of 0.002 awarded for producing any emotion word, acknowledging emotion labelling even when the child’s response it did not match adult labels (this value represents the rounded mean between the frequency score assigned for no emotion label and the score assigned for labels produced by only one adult); and (c) a vignette difficulty score capturing variability in adult responses, quantified using entropy^37^. Lower entropy values indicate high agreement (i.e., easier vignettes), whereas higher values indicate more variable labelling and thus greater difficulty. These components were combined into a weighted sum score (max. 5.00) across all vignettes, such that higher values reflected a more adult-like, i.e., differentiated precise, and contextually appropriate use of emotion words. Full technical details, including formulae and calculation examples, are provided in Streubel et al.^20^. Conceptually, this scoring approach captures emotion-specific-vocabulary depth as a qualitative characteristic of children’s emotion-specific vocabulary, reflecting how precisely and context-appropriate children apply the emotion words available to them. In line with the definition outlined in the Introduction, emotion-specific vocabulary size refers to the number of distinct emotion words a child produces, whereas depth reflects how closely a child’s usage approximates adult-like differentiation of emotion concepts across situations. Accordingly, depth is inherently constrained by vocabulary size, as children can only demonstrate depth for emotion concepts they already know.

#### Emotion knowledge

We assessed emotion recognition and knowledge of emotion regulation strategies using subscales from the Intelligence and Development Scales 2 (IDS-2)^62^. The emotion recognition subscale includes 10 photos showing prototypical facial expressions of joy, anger, fear, sadness, and surprise (each posed by a girl and a boy). Children named the emotions, scoring one point per correct identification (max. 10). The emotion regulation subscale, embedded in the emotion recognition task, followed the presentation of three photos (anger, fear, and sadness). Children were presented with two different scenarios each describing a child experiencing the depicted emotion (e.g., “Imagine this boy is angry because another child broke his toy”). Children were asked to advise how the boy/girl could stop feeling that emotion. Scoring awarded two points for adaptive strategies (e.g., problem-focused coping, distraction, reappraisal), zero points for maladaptive strategies (e.g., aggressive behavior, self-deprecation, perseveration), and one point for other strategies (seeking support, expressing emotions), with a maximum of 12 points (3 emotions × 2 scenarios × 2 points) (for more details, please refer to Grob & Hagmann-von Arx^62^.

#### Regulation of emotional expressivity

Children completed the Balloons Game, a computer task assessing regulation of emotion-expressive behavior^50,51^. In this game, blue, red, and yellow balloons floated upward, and children were instructed to pop as many yellow balloons as possible by clicking with a mouse. Balloon speed was individually calibrated during a 90-second phase, to allow each child to pop all yellow balloons and win the game. The game consisted of eight 40-second trails with 10-second breaks. *Success trials* (S), allowed children to pop all or almost all yellow balloons, ending with a cheerful melody and happy emoji to induce joy and pride. In *failure trials* (F), mouse control was manipulated in the last 10 s, preventing further popping and causing children to lose the trial. Failure trials ended with an error sound and sad emoji to induce frustration and anger. Each child played two conditions: a *non-regulation* condition, with no specific instructions, and a *regulation* condition where children were asked to downregulate any facial or behavioral expressions so that the investigator’s “colleagues who will watch the tape will not be able to see whether you are winning or losing the game”. The regulation condition always followed the non-regulation condition to avoid carryover effects^51^. Both conditions started with two success trials, followed by six trials in a fixed pseudo-randomised order (S-S-F-S-F-S-F-S). Sessions were recorded via a Logitech HD 1080 webcam.

Facial expressions during both conditions were coded using FaceReader 9.0 (Noldus, Leesburg, VA Inc)^52^, an automated analysing system based on FACS (Facial Action Coding System) that detects facial muscle contractions and scores the intensity (0 = non to 1 = intense) for six emotions: happiness, sadness, surprise, anger, fear, and disgust. FaceReader has demonstrated high validity, reliability^63,64^, and accuracy in comparison to manually coded FACS scores^65,66^.

For each condition six trials (three success and three failure) were analysed, excluding the first and last trial to ensure a balanced success-failure-trial-ratio. We coded a 20-second window around each trials end (10 s before and after the success/failure emoji and sound), capturing the immediate emotional response^50^. Coding was done at 20-millisecond intervals (five frames per second), yielding 100 frames per trial and 300 frames per emotion and condition.

Two raw expressivity scores per condition were computed: negative expressivity during failure (mean intensity of anger, sadness, fear, and disgust across all 3 failure trials per condition) and positive expressivity during success (mean intensity of happiness across all 3 success trials per condition). Two emotion regulation indices were calculated as the ratio of expressivity in the regulation condition over the non-regulation condition, separately for negative and positive expressivity: *ER negative* (negative expressivity during failure in the regulation condition divided by negative expressivity during failure in the non-regulation condition) and *ER positive* (positive expressivity during failure in the regulation condition divided by positive expressivity during failure in the non-regulation condition). The indices were multiplied by − 1 so that higher scores indicate greater emotion regulation^50^. The following example illustrates the scoring procedure. In the event of a child showing an average negative expressivity of 0.42 (mean intensity of anger, sadness, fear, and disgust) across the three failure trials in the non-regulation condition and an average negative expressivity of 0.30 in the regulation condition, the ER negative index is calculated as 0.30/0.42 × (–1) = − 0.71. After multiplication by (− 1), values greater than (− 1) indicate a reduction in negative facial expressions from the non-regulation to the regulation condition, whereas values smaller than (− 1) indicate an increase of negative facial expressions from the non-regulation to the regulation condition. The same computational procedure was applied to positive expressivity during success trials.

### Data analysis

To address research question 1, we conducted multiple stepwise regression analyses to examine the relative contributions of emotion-specific and general vocabulary to individual differences in emotion knowledge, using children’s scores of emotion recognition and knowledge of emotion regulation strategies as the dependent variables.

For research question 2, we first tested whether children showed reduced expressivity in the regulation condition of the Balloons Game, as an indicator of responsiveness to the regulatory request. To this end, a repeated measures ANOVA was conducted on raw expressivity scores across the non-regulation and regulation condition. We then conducted multiple stepwise regression models to test whether ER positive and ER negative as dependent measures were associated with both general and emotion-specific vocabulary. Exploratory data analysis revealed potential outliers (> 3 SD from the mean) in both ER indices. To minimize their influence, all regression models were estimated using robust regression via the *lmrob()* function in R, which applies M-estimation using iteratively reweighted least squares (IRWLS)^67^. This method employs a weighting and discounting system for observations based on their outlier status and diverges from the conventional ordinary least squares (OLS) approach^68^. We base our conclusions for research question 2 on the robust regression model. For transparency, results from the OLS regression are reported in the Supplementary Information (Table S1).

To explore research question 3, we tested the mediating role of emotion knowledge (i.e., emotion recognition and knowledge of ER strategies) in the relationship between vocabulary skills and children’s regulation of emotion-expressive behavior, following the steps proposed by Baron and Kenny^69^. Specifically, we examined (1) whether vocabulary measures predicted the proposed mediators (emotion recognition and knowledge of emotion regulation strategies), (2) whether vocabulary measures predicted regulation of emotional expressivity, and (3) whether the mediators predicted ER. In line with more recent methodological approaches^47–49^, we did not consider a significant total effect, i.e., a direct association between vocabulary measures and ER as a necessary precondition for testing a mediation effect. Instead, we assumed only the associations between the independent variables (vocabulary measures) and the mediators (emotion knowledge subscales) as well as between the mediators and the dependent variables (ER indices) as essential conditions for further mediation analyses. If these conditions were met, we tested whether including the mediators reduced the strength of the vocabulary–ER relationship.

In all aforementioned regression models, predictors were z-standardized to facilitate the interpretation of main and interaction effects^70^.

## Supplementary Information

Below is the link to the electronic supplementary material.

Supplementary Material 1

## Data Availability

The data generated and analysed during the present study are available in the OFS repository [https://osf.io/7m6py].
