# Supplementary material for: Emotion-specific vocabulary is associated with preschoolers’ emotion knowledge and behavioral emotion regulation
Source: Sci Rep. 2026 Feb 6;16:5414. doi: 10.1038/s41598-026-38847-3 (PMC12887049; doi:10.1038/s41598-026-38847-3)
Supplement: Supplementary file 1 — Supplementary Material 1 [file 41598_2026_38847_MOESM1_ESM.pdf]

## Supplementary Information

Title of the manuscript: *Emotion-specific vocabulary is associated with preschoolers' emotion knowledge and behavioral emotion regulation*

Authors: Berit Streubel, Nadia Khammous, Henrik Saalbach, Catherine Gunzenhauser

**Table S1. Stepwise Multiple Regression Analyses Predicting Children's Regulation of Emotional Expressivity from General and Emotion-specific Vocabulary.**

| Predictors (*z*-standardized) | Regulation of positive expressivity | Regulation of negative expressivity |
|---|---|---|
| Model 1 | | |
| General vocabulary | .453 | -.050 |
| $R^2$ | .012 | .003 |
| *F* | 2.33 | .503 |
| Model 2a | | |
| General vocabulary | $.619^{*}$ | -.054 |
| Emotion-specific vocabulary size | -.585 | .015 |
| $R^2$ | .029 | .002 |
| *F* | 2.99 | .269 |
| Model 2b | | |
| General vocabulary | .302 | -.048 |
| Emotion-specific vocabulary depth | .550 | -.008 |
| $R^2$ | .028 | .003 |
| *F* | 2.78 | .256 |
| Model 3 | | |
| General vocabulary | .469 | -.052 |
| Emotion-specific vocabulary | | |
| Size | $-.948^{**}$ | .021 |
| Depth | $.921^{**}$ | -.016 |
| $R^2$ | .068 | .003 |
| *F* | $4.70^{**}$ | .192 |
| Model 4 | | |
| General vocabulary | .457 | -.052 |
| Emotion-specific vocabulary | | |
| Size | $-.923^{**}$ | .022 |
| Depth | $1.048^{**}$ | -.009 |
| Size x depth | .462 | .028 |
| $R^2$ | .081 | .004 |
| *F* | 4.23 | .184 |

*Note.* Standardized regression coefficients (*β*), $R^2$, F-statistics, and significance levels are reported. $^{**}$ $p \leq .01$. $^{***}$ $p \leq .001$
